# Tbx20 Is an Essential Regulator of Embryonic Heart Growth in Zebrafish

**DOI:** 10.1371/journal.pone.0167306

**Published:** 2016-12-01

**Authors:** Steffen Just, Linda Raphel, Ina M. Berger, Anja Bühler, Mirjam Keßler, Wolfgang Rottbauer

**Affiliations:** 1 Molecular Cardiology, Department of Medicine II, University of Ulm, Ulm, Germany; 2 Department of Medicine II, University of Ulm, Ulm, Germany; Academia Sinica, TAIWAN

## Abstract

The molecular mechanisms that regulate cardiomyocyte proliferation during embryonic heart growth are not completely deciphered yet. In a forward genetic N-ethyl-N-nitrosourea (ENU) mutagenesis screen, we identified the recessive embryonic-lethal zebrafish mutant line *weiches herz* (*whz*). Homozygous mutant *whz* embryos display impaired heart growth due to diminished embryonic cardiomyocyte proliferation resulting in cardiac hypoplasia and weak cardiac contraction. By positional cloning, we found in *whz* mutant zebrafish a missense mutation within the T-box 20 (Tbx20) transcription factor gene leading to destabilization of Tbx20 protein. Morpholino-mediated knock-down of Tbx20 in wild-type zebrafish embryos phenocopies *whz*, indicating that the *whz* phenotype is due to loss of Tbx20 function, thereby leading to significantly reduced cardiomyocyte numbers by impaired proliferation of heart muscle cells. Ectopic overexpression of wild-type Tbx20 in *whz* mutant embryos restored cardiomyocyte proliferation and heart growth. Interestingly, ectopic overexpression of Tbx20 in wild-type zebrafish embryos resulted, similar to the situation in the embryonic mouse heart, in significantly reduced proliferation rates of ventricular cardiomyocytes, suggesting that Tbx20 activity needs to be tightly fine-tuned to guarantee regular cardiomyocyte proliferation and embryonic heart growth *in vivo*.

## Introduction

The adult mammalian heart largely lacks regenerative capacity, making it refractory to structural and functional recovery after acute or chronic damage [1–3]. Intriguingly, in the embryonic and neonatal mammalian heart, there seems to be still pronounced plasticity and adaptive cell proliferation, which however is gradually lost during early childhood, rendering cardiomyocytes mostly post-mitotic in adulthood [4, 5]. By contrast and similar to the situation in the embryonic and neonatal mammalian heart, both, embryonic and adult zebrafish cardiomyocytes are not post-mitotic, warranting the efficient regenerative proliferation of pre-existing, spared cardiomyocytes after myocardial injury [6]. To which extent molecular programs used during cardiac development are re-deployed during heart regeneration is incompletely understood. Furthermore, the molecular mechanisms that regulate cardiomyocyte proliferation during embryonic heart development in vertebrates are not thoroughly understood. Recently, large-scale N-ethyl-N-nitrosourea (ENU)-mutagenesis screens for cardiac hypo- and hyperplasia mutants in zebrafish were shown to be suitable to identify novel regulators of embryonic cardiac growth. In this context, an activating mutation of the transcriptional repressor RuvB-like AAA ATPase 2 Reptin in the zebrafish mutant *liebeskummer* increases embryonic cardiomyocyte proliferation, at least in part via the modulation of β-catenin signaling [7]. Furthermore, the calcium channel voltage-dependent, L type, alpha 1C subunit (CACNA1C) in the zebrafish mutant *island beat* was also shown to regulate heart growth. [8]. Here, disruption of CACNA1C and thereby altered calcium signaling within embryonic cardiomyocytes led to hypoplastic hearts. Moreover, the zebrafish SWI/SNF related, matrix associated, actin dependent regulator of chromatin, subfamily a, member 4 (SMARCA, BRG1; zebrafish mutant *brg1*^*s481*^) was found to be critical for cardiogenesis in zebrafish and to play an essential role in the regulation of embryonic cardiomyocyte proliferation *in vivo* [9].

The family of T-box (Tbx) transcription factors plays a crucial role during embryonic development in all metazoans. Especially, T-box factors such as Tbx1, Tbx2, Tbx3, Tbx5, Tbx18 and Tbx20 are described to be involved in virtually all steps of cardiogenesis and mutations are associated with developmental syndromes and cardiac abnormalities like DiGeorge syndrome (Tbx1) or Holt-Oram syndrome (Tbx5) [10–12]. Additionally, human Tbx20 mutations were associated with atrial septal defects and cardiomyopathy, whereas Tbx20 null mice die at mid-gestation due to the lack of proper heart chamber formation and cardiac hypoplasia [13, 14]. In zebrafish, Morpholino-mediated knock-down of Tbx20 was found to cause defective heart chamber morphology and impaired development of the atrio-ventricular boundary in embryonic hearts [15].

Here, we show that embryonic cardiomyocytes in the ENU-induced recessive lethal zebrafish mutant *weiches herz* (*whz*^m245^) fail to proliferate, leading to severe cardiac hypoplasia and finally heart failure. By positional cloning, we demonstrated that a missense mutation in the zebrafish *tbx20* gene, leading to the destabilization of Tbx20 protein, is responsible for the *whz* phenotype. Consistently, embryonic cardiac hypoplasia can also be induced by targeted knock-down of Tbx20, whereas cardiomyocyte proliferation can be restored in *whz* mutant hearts by ectopic overexpression of wild-type Tbx20. Remarkably, ectopic overexpression of Tbx20 in wild-type zebrafish embryos resulted in reduced proliferation rates of ventricular cardiomyocytes, suggesting that Tbx20 levels need to be tightly regulated during embryonic heart growth. Taken together, our findings demonstrate a pivotal role of Tbx20 in the regulation of cardiomyocyte proliferation in the embryonic zebrafish heart.

## Materials and Methods

### Zebrafish strains and injection procedures

All procedures and experiments in this study were carried out after appropriate institutional approvals (Tierforschungszentrum (TFZ) Ulm University, No. 0183), which conform to the EU Directive 2010/63/EU.

Microinjections were performed into fertilized zebrafish oocytes at the 1–2-cell stage, using pulled glass capillaries (World Precision Instruments, Sarasota, FL) and a Microinjector (Eppendorf, Hamburg, Germany). Embryos were then allowed to develop at 28.5°C until the indicated stages. To inhibit pigmentation, 0.003% 1-phenyl-2-thiourea was added to the regular embryo medium E3 (5 mM NaCl, 0.17 mM KCl, 0.33 mM CaCl_2_, 0.33 mM MgSO_4_ dissolved in water).

For the Morpholino-modified antisense oligonucleotide (MOs; Gene Tools, LLC, Oregon, USA) injection procedures, TL wild-type strains were used. MOs were directed either against the translational start site (MO1-*tbx20*: GGGAAGAGGTGTACTCCATGACGCT) or against the splice donor site of exon 3 (MO2-*tbx20*: AGAAACAAATTCCTACCTAGCAGGT) of zebrafish *tbx20*. Five base mismatch Morpholino-modified antisense oligonucleotides were injected at the same concentration as negative controls (MO1-ctrl: GTGTAGTGGTTTACTCCTTGACGCT and MO2-ctrl: GCGTAGTGGTGTACTCGATGACCCT) (GENETOOLS, LLC).

For rescue experiments, sense capped mRNA of *Myc*-tagged zebrafish *tbx20* was synthesized using the mMESSAGE mMASCHINE system (Ambion, Cat. No. AM1340).

### Isolation of zebrafish protein lysates and Western blot analysis

For each of the four independent Western blot experiments, 100 embryonic hearts from wild-type and *whz* mutant zebrafish, respectively, expressing GFP exclusively in cardiomyocytes (Tg(cmlc2:rasEGFP)) were dissected out at 72 hpf and protein lysates were prepared. For Western blot analysis each protein lysate was boiled in 3x Laemmli Buffer and loaded on a precast 10% SDS gel (Bio-Rad). Proteins were separated by SDS-PAGE and transferred to polyvinylidene fluoride (PVDF) membrane. After blocking in 5% milk powder in TBST for 2h at RT, the membrane was incubated with the primary antibody over night at 4°C. The secondary antibodies were incubated for 2h at RT [16].

The following primary antibodies were used: Tbx20 primary antibody (1:1000, abcam; ab93058). Signals were detected by chemiluminescence (anti-rabbit IgG HRP-linked, Cell Signaling), using a luminescent image analyzer (Image Quant Las4000 mini). In-vitro synthesized Tbx20 protein was generated following standard protocols (TnT Kit, Promega) and used as positive control for western blot analysis. Western blots were quantified by using Image Quant LAS4000 software.

### *In situ* hybridization and Histology

A standard protocol was followed for *in situ* hybridization of embryos after fixation and dechorionation at the indicated stages. Probes were prepared by *in vitro* transcription. Whole-mount *in situ* hybridization was performed using Digoxigenin labelled antisense RNA probe and visualized using anti-Digoxigenin Fab fragments conjugated with alkaline phosphatase (Roche Molecular Biochemicals) as described [17, 18]. Embryos were processed and hybridized as described [19], except that 10 mg/ml of proteinase K in PBS/0.1% Tween-20 was used for 10 to 30 minutes depending on the age of the collected embryos.

For histology, zebrafish embryos were fixed in 4% paraformaldehyde and dehydrated prior embedding in JB-4 (Polysciences). 5-μm sections were cut using Leica RM2255 microtome (Leica, Wetzlar, Germany), dried, and stained with Hematoxylin and Eosin as described [20]. Still images were taken with an Olympus SZX 16 and Zeiss stereomicroscope (AxioSkop 2 plus).

### Genetic mapping, positional cloning and mutation detection

DNA from 48 *whz* mutants and 48 wild-type siblings was pooled and bulked segregation analysis was performed. The critical genomic interval for *whz* was defined by genotyping 1750 mutant embryos for polymorphic markers in the area. The *whz* locus was restricted to three overlapping bacterial artificial chromosomes (BAC) zC215K15, zC78C9 and zC281F5. Further recombination analyses using single nucleotide polymorphisms (SNPs) and simple sequence length polymorphisms (SSLP) derived from sequence of the overlapping BAC clones, the *whz* mutation interval could be restricted to a 89 kb region on BAC zC215K15 that contains 2 open reading frames encoding the proteins HERPUD family member 2 (herpud2; NP_956482) and the zebrafish T-box transcription factor Tbx20 (NP_571581). The stop codon mutation was detected by sequencing of the entire coding region of zebrafish *herpud2* and *tbx20* from wild-type and *whz* mutant cDNA.

### RNA extraction and quantitative real-time PCR

For each the 4 biological replicates, a pool of 25 embryos were collected at 72 hpf. RNA extraction was carried out by RNeasy® Mini Kit (Qiagen) according to the manufacturer’s instructions. Total RNA (1 μg) was reverse transcribed to produce cDNA using Superscript III reverse transcriptase (Life Technologies).

Quantitative real-time PCR was carried out according to standard protocols using SYBR-Green (Roche) on a Roche LightCycler 480 II. Three different house-keeping genes, rpl13a (fw: 5’-TCTGGAGGACTGTAAGAGGTATGC-3’; rv: 5’- AGACGCACAATCACAATCTTGAGAGCAG-3’), β-actin2 (fw: 5’-GCAGAAGGAGATCACATCCCTGGC-3’; rv: 5’-CATTGCCGTCACCTTCACCGTTC-3’) and 18s (fw: 5’-CACTTGTCCCTCTAAGAAGTTGCA-3’; rv: 5’-GGTTGATTCCGATAACGAACGA-3’) were used as reference genes for the normalization against *tbx20* (fw: 5’-CTAGGTTATATGTTCATCCAGATTCG-3’; rv: 5’-GCATGGAGTTGAGGATGATATG-3’).

### Terminal deoxynucleotidyl transferase Dig-dUTP nick end-labeling (TUNEL)

To analyze cardiomyocyte apoptosis, TUNEL stainings were performed on zebrafish embryos at 72 hpf according to the manufacturer’s instructions (Merck Millipore). For histology, the TUNEL stained zebrafish embryos were dehydrated prior to embedding in JB-4 (Polysciences). 5-μm sections were cut using a Leica RM2255 microtome (Leica, Wetzlar, Germany), dried, and stained with Hematoxylin and Eosin as described [21]. Images were recorded using a Zeiss Axioskop 2 plus.

### Isolation of embryonic hearts and proliferation assay

Proliferating cardiomyocytes in zebrafish hearts at 72 hpf were counted upon the detection of DNA synthesis by the incorporation of 5-ethynyl-2'-deoxyuridine (EdU) using the Click-iT EdU Imaging Kit (Life Technologies, Carlsbad, CA, USA). Cardiomyocyte nuclei were counterstained using an antibody against Mef-2 (Santa Cruz Biotechnology C-21 HO310) at a dilution of 1:200. Anti-rabbit Alexafluor 555 antibodies were used to detect the fluorescence by an iMIC digital microscope (Till Photonics) or a confocal microscope (Leica TCS SP8).

### Statistical analysis

All graphs and statistical analyses were prepared with the help of Prism4 (GraphPad software). If not further specified, results are expressed as mean ± S.D. Analyses were performed using nonparametric Mann–Whitney tests and a value of *P*<0.05 was accepted as statistically significant.

## Results

### Embryonic cardiomyocyte proliferation is reduced in *weiches herz* embryos

**Fig 1:**
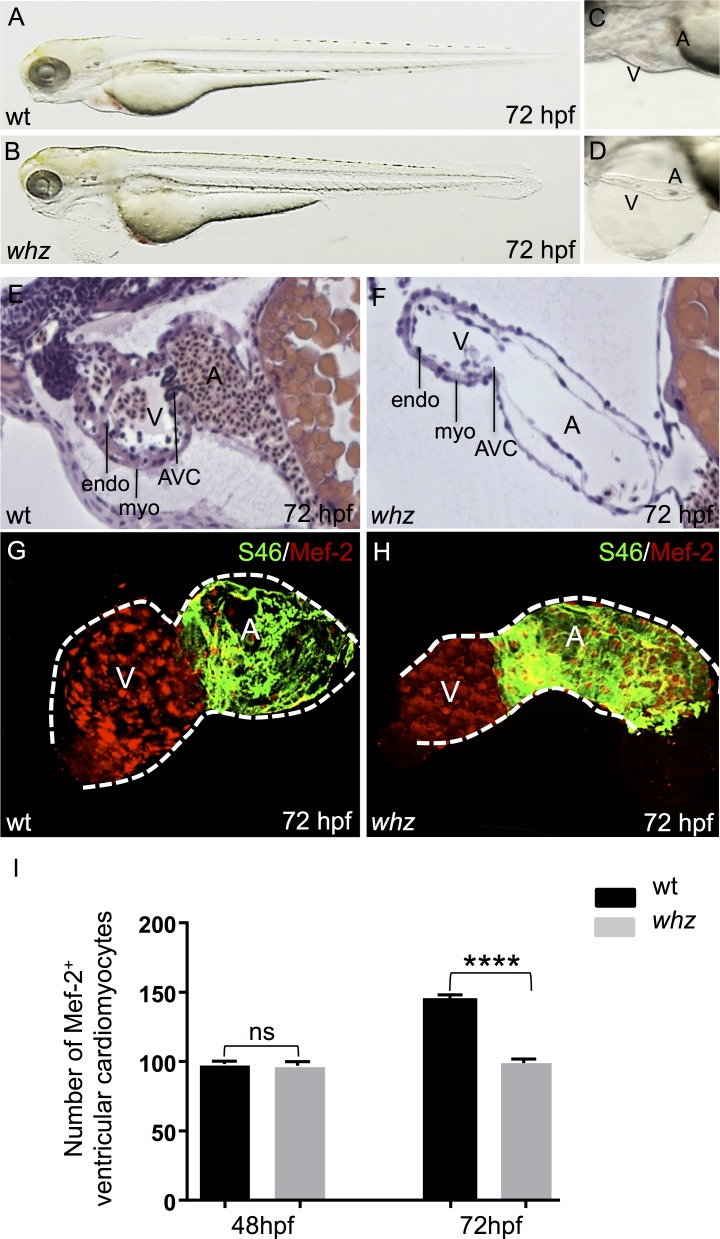
Effects of the *whz* mutation on embryonic heart morphology and growth. **(A-D)** Lateral view of wild-type (wt; **A, C**) and *whz* mutant (**B, D**) embryos at 72 hours post fertilization (hpf). *Whz* mutants show pericardial edema, blood congestion at the cardiac inflow tract and stretched heart chambers. **(E, F)** Hematoxylin and Eosin staining of sagittal histological sections of wt **(E)** and *whz* mutant **(F)** hearts at 72 hpf. Similar to wild-types, in *whz* atria and ventricles, myocardial (myo) and endocardial (endo) cell layers are clearly defined and separated by an atrio-ventricular canal (AVC). In contrast to wild-type hearts, *whz* mutant ventricles appear small and the myocardium monolayered. **(G-I)** Dissected wt **(G)** and *whz* mutant **(H)** hearts at 72 hpf, stained with a cardiomyocyte-specific MEF-2 antibody (nuclei; red) and co-stained with S46, exclusively marking atrial cardiomyocytes (green)**. (I)** *whz* mutant hearts show significantly reduced ventricular cardiomyocytes at 72 hpf (sib: 144.2±10 SD and *whz*: 94.9±10 SD, n = 10; p<0.0001), whereas cardiomyocyte numbers are comparable between wt and *whz* ventricles at 48 hpf (wt: 93.4±10 SD and *whz*: 88.2±10 SD, n = 10; p>0.05).

In search for novel regulators of embryonic cardiomyocyte proliferation, we identified here by visual screening the “small heart” zebrafish mutant *weiches herz* (*whz*^m245^) [22] (Fig 1A and 1B). Homozygous *whz* mutant embryos display small and only weakly contracting hearts and die 5 days post-fertilization (dpf) (Fig 1A–1D).

By 72 hpf, myocardial and endocardial cell layers are clearly defined in *whz* mutant hearts but the ventricular chambers remain small and the myocardium single-layered (Fig 1E and 1F). Starting at 48 hpf, ventricular myocardium in zebrafish usually thickens by the addition of new cardiomyocytes [7, 8]. To assess whether the observed “small heart” phenotype is due to reduced cardiomyocyte numbers in *whz* mutant hearts, we evaluated dissected wild-type and *whz* mutant hearts stained with antibodies against MEF-2 (Myocyte Enhancer Factor-2), known to mark nuclei of cardiomyocytes, and S46, exclusively labeling atrial cardiomyocytes, at 48 and 72 hpf. At 72 hpf, we found significantly less ventricular cardiomyocytes in *whz* mutants compared to wild-type littermates (sib: 144.2±10 SD and *whz*: 94.9±10 SD, n = 10 hearts, 3 independent experiments; p<0.0001) (Fig 1G–1I), whereas at 48 hpf cardiomyocyte numbers in *whz* mutant ventricles were not reduced compared to control embryos (wt: 93.4±10 SD and *whz*: 88.2±10 SD, n = 10 hearts, 3 independent experiments; p>0.05) (Fig 1I), suggesting that *weiches herz* interferes with cardiac growth after 48 hpf.

**Fig 2:**
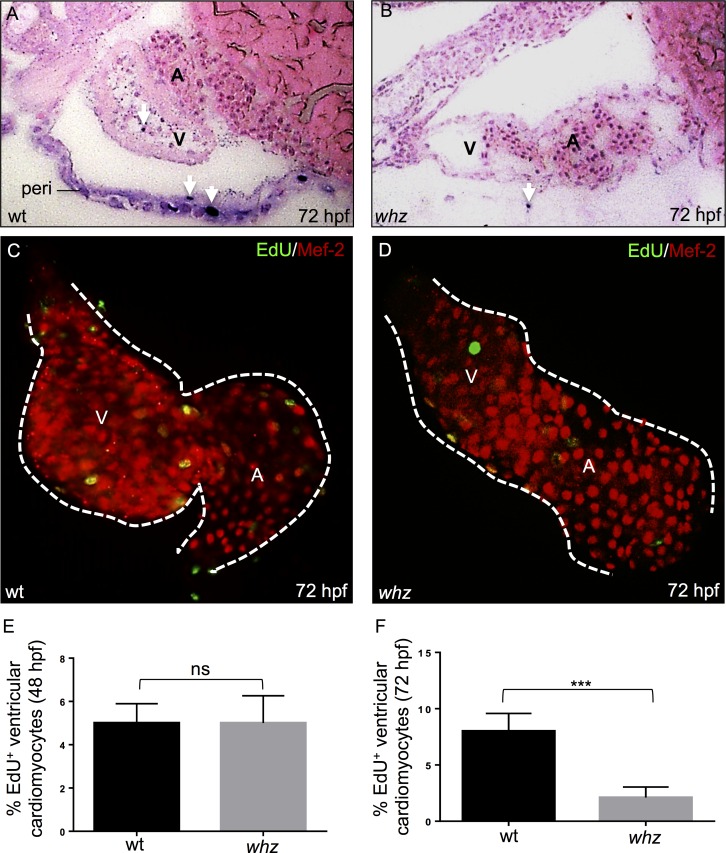
The *whz* mutation interferes with cardiomyocyte proliferation. **(A, B)** TUNEL stainings of embryonic zebrafish hearts at 72 hpf show no difference in the number of apoptotic cardiomyocytes in wt **(A)** and *whz* **(B)** mutant embryos. TUNEL positive cells in the pericardium (peri) and ventricles are marked by arrows. **(C-F)** Dissected wt **(C)** and *whz* mutant **(D)** hearts at 72 hpf, stained against MEF-2 (red) after incorporation of 5-ethynyl-2'-deoxyuridine (EdU; green) to visualize cardiomyocyte proliferation. At 48 hpf, proliferation of ventricular cardiomyocytes appears unaltered between wt and *whz* mutant hearts (sib: 5±2% SD and *whz*: 4±2% SD, n = 10; p>0.05) **(E)**, whereas cardiomyocyte proliferation in *whz* mutant ventricles is significantly reduced compared to wt at 72 hpf (sib: 8±2% SD, *whz*: 2±2% SD, n = 10, p = 0.0001) **(F)**.

To investigate whether increased cardiomyocyte apoptosis is the cause for cardiac hypoplasia in *whz* at 72 hpf, we conducted TUNEL assays, performed serial histological sections and quantified the number of apoptotic cardiomyocytes. We found no increase in the number of apoptotic cardiomyocytes in *whz* mutant hearts compared to wild-type littermates (wt = 0.40 ± 0.16; *whz* = 0.30±0.15, n = 10 hearts, 3 independent experiments; p = 0.6601) (Fig 2A and 2B).

Next, to assess the number of proliferating cardiomyocytes in wild-type and *whz* mutant embryos at 48 and 72 hpf, EdU-stainings marking cells in the S-phase of the cell cycle in combination with Mef-2 antibody staining were performed (Fig 2C and 2D). At 72 hpf, quantification of EdU-positive cardiomyocytes revealed significantly reduced numbers of proliferating cardiomyocytes in *whz* mutant hearts compared to wild-type littermates (sib: 8 ± 2% SD, *whz*: 2 ± 2% SD, n = 10 hearts, 3 independent experiments; p = 0.0001) (Fig 2F), whereas the percentage of proliferating cardiomyocytes at 48 hpf did not differ between wild-type and *whz* mutant embryos (sib: 5±2% SD and *whz*: 4±2% SD, n = 10 hearts, 3 independent experiments; p>0.05) (Fig 2E).

These findings indicate that rather impaired cardiomyocyte proliferative growth accounts for the small heart phenotype in *whz* mutant zebrafish embryos and not increased cardiomyocyte apoptosis.

### *Weiches herz (whz*^m245^) encodes zebrafish *t-box transcription factor 20 (tbx20)*

**Fig 3:**
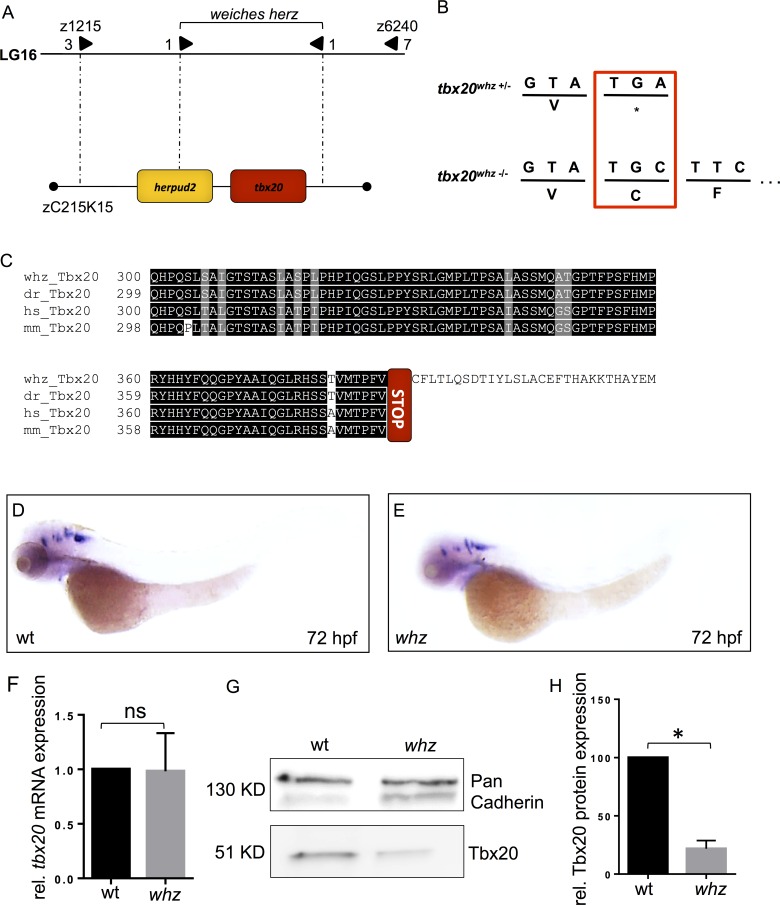
*whz* encodes *Tbox transcription factor 20 (tbx20)*. **(A)** Integrated genetic and physical map of the *whz* locus on zebrafish chromosome 16. The *whz* mutation interval is flanked by the microsatellite markers z1215 and z6240 and encodes 2 open reading frames, zebrafish *tbx20* and *herpud2*. **(B)** Missense mutation of Adenine to Cytosine of the stop codon of *tbx20* results in the loss of the original stop codon and the termination of *tbx20* transcription after 87 additional nucleotides. **(C)** Partial amino acid alignment of the C-terminus of zebrafish *whz* and wt as well as human and murine Tbx20. Tbx20 is highly conserved cross-species and the *whz* mutant Tbx20 protein is extended by 29 additional amino acids. **(D, E)** *Tbx20*-specific whole-mount antisense RNA *in situ* hybridization detects unaltered expression of zebrafish *tbx20* in *whz* mutant embryos compared to wild-types. **(F)** Quantitative RT-PCR analysis showing similar relative mRNA levels of *tbx20* in wt and *whz* embryos at 72 hpf (n = 4; p = 0.5957). **(G, H)** Tbx20 protein levels are significantly reduced in *whz* mutant embryos compared to wild-type littermates (n = 4; p = 0.0286).

To reveal the ENU-induced genetic defect in *whz*^m245^ mutant embryos, we performed a genome-wide study of microsatellite marker segregation by bulked segregant analysis and located *whz* in-between the microsatellite marker Z1215 and Z6240 on chromosome 16. Genetic fine-mapping by recombination analysis of 1750 *whz* mutant embryos restricted the *whz* locus to three overlapping bacterial artificial chromosomes (BAC) zC215K15, zC78C9 and zC281F5. Further recombination analyses using single nucleotide polymorphisms (SNPs) and simple sequence length polymorphisms (SSLP) derived from sequence of the overlapping BAC clones, the *whz* mutation interval was restricted to a 89kb region on BAC zC215K15 (Acc.: CR391962) that contains 2 open reading frames encoding the proteins HERPUD family member 2 (herpud2; NP_956482) and the zebrafish T-box transcription factor Tbx20 (NP_571581) (Fig 3A).

To finally identify the ENU-induced mutation in *whz*, we sequenced the entire coding sequences of zebrafish *herpud2* and *tbx20* from wild-type and *whz* mutant cDNA. We found the *whz* mutation to be a transversion of nucleotide Adenine to Cytosine at codon 1225 of zebrafish tbx20 (Fig 3B), whereas in *herpud2* no mutation was detected by cDNA sequencing. Interestingly, the identified mutation is predicted to change the *tbx20* stop codon into the amino acid cysteine (Fig 3B) thereby resulting in prolongation of the *whz* mutant Tbx20 protein by 29 amino acids (Fig 3C).

To test whether the identified loss-of-stop mutation in *whz* results in degradation of *whz* mutant *tbx20* mRNA, we performed whole-mount antisense RNA *in situ* hybridizations on wild-type and *whz* mutant embryos at 72 hpf using *tbx20*-specific riboprobes. We found no alterations in *tbx20* mRNA levels and location between *whz* mutants and wild-type littermates (Fig 3D and 3E). To confirm these findings from whole-mount antisense *in situ* hybridizations, we next performed quantitative RT-PCR analyses of *tbx20* mRNA levels in wild-type and *whz* mutant embryos at 72 hpf. We found that *tbx20* mRNA levels were not affected by the *whz* mutation (n = 4; p = 0.5957), suggesting that *whz* mutant *tbx20* mRNA is not degraded by nonsense-mediated RNA decay (Fig 3F).

Next, to assess whether the predicted Tbx20 protein elongation in *whz* mutant embryos destabilizes Tbx20 proteins, we conducted Western blot analyses using a Tbx20-specific antibody and found that Tbx20 protein levels were significantly reduced (n = 4 experiments; p = 0.0286) (Fig 3G and 3H). These findings suggest that the *whz* loss-of-stop mutation and the addition of 29 amino acids at the C-terminus of Tbx20 leads to a destabilization and degradation of the protein.

### Targeted knock-down of Tbx20 in wild-type zebrafish leads to inhibition of embryonic cardiomyocyte proliferation

**Fig 4:**
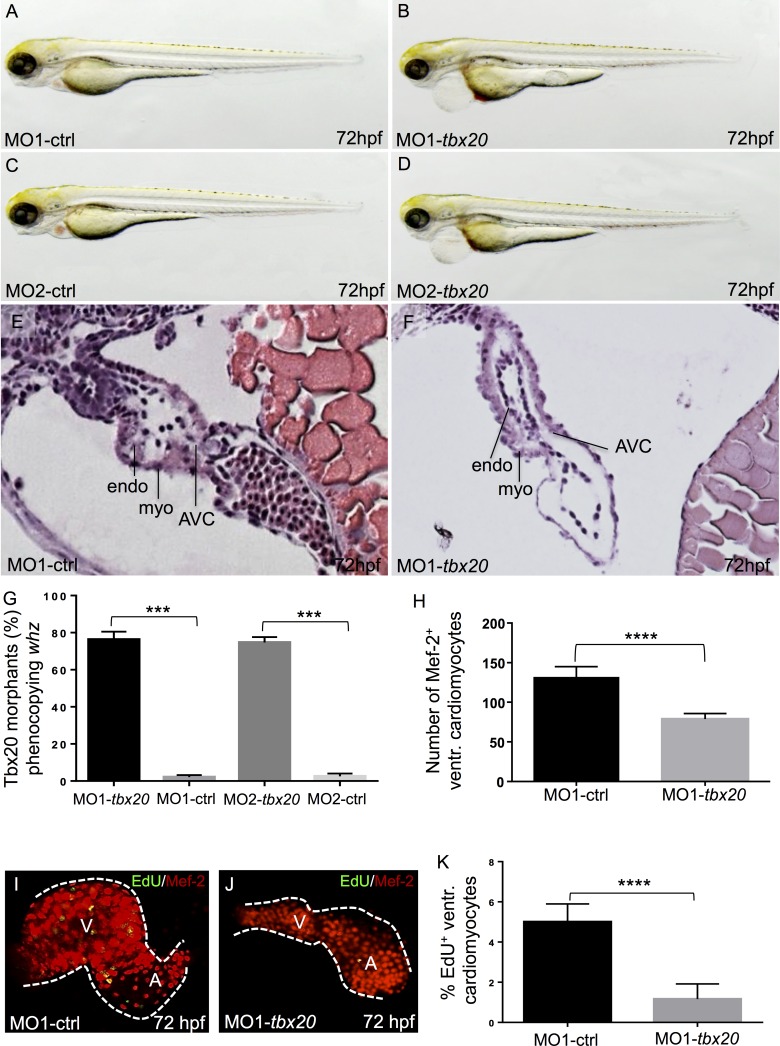
Knock-down of zebrafish *tbx20* phenocopies the *whz* mutant phenotype. **(A-D)** Lateral view of wild-type embryos injected with zebrafish *tbx20*-specific control Morpholinos (MO-ctrl) **(A, C)** and *tbx20* start and splice Morpholinos (MO-*tbx20*) **(B, D)** at 72 hpf, respectively. Knock-down of *tbx20* phenocopies the *whz* mutant phenotype, whereas injection of the same amount of specific-control Morpholinos does not affect heart growth. **(E, F)** Hematoxylin and Eosin staining of sagittal histological sections of MO-ctrl **(E)** and MO-*tbx20* **(F)** injected hearts at 72 hpf. In contrast to control hearts, *tbx20* morphant ventricles appear small and the myocardium monolayered. **(G)** 78% (MO1-*tbx20*) and 76% (MO2-*tbx20*) of the injected embryos are indistinguishable from *whz* mutant embryos. **(H)** *Tbx20* morphant hearts show significantly reduced ventricular cardiomyocytes at 72 hpf (MO1-control: 130.7±10 SD, MO1-*tbx20*: 86.5±10 SD, n = 10; p = 0.0001). **(I-K)** Cardiomyocyte proliferation in *Tbx20* morphant ventricles is significantly reduced compared to controls at 72 hpf (MO1-control: 6±2% SD, MO1-*tbx20*: 1±2% SD, n = 10; p = 0.0001).

Our Tbx20 Western blot analyses imply that rather loss of Tbx20 function than a dominant-negative effect of elongated Tbx20 appears to cause the *whz* ventricular hypoplasia phenotype. To further validate the finding that ventricular hypoplasia in *whz* mutants is caused by loss of Tbx20 function, we injected Morpholino-modified antisense oligonucleotides (MOs), either directed against the translational start site (MO1-*tbx20*) or the splice donor site of exon 3 (MO2-*tbx20*) of zebrafish *tbx20* into wild-type zebrafish embryos at the one-cell stage. When injected with 2.5 ng of MO1-*tbx20* or 3.1 ng of MO2-*tbx20*, 78% and 76% of the injected embryos (n = 150, respectively) displayed the *whz* mutant phenotype (Fig 4B, 4D and 4G) indicating a mono-layered, small ventricle (Fig 4F). Injection of the same amount of the corresponding five base pair mismatch MOs as control did not result in any pathology (Fig 4A, 4C, 4E and 4G).

Next, we measured cardiomyocyte numbers in Tbx20 morphant zebrafish hearts at 72 hpf. Similar to the situation in *whz* mutant hearts, we found significantly lower amounts of ventricular cardiomyocytes in Tbx20 morphants compared to MO-control injected embryos (MO1-control: 130.7±10 SD, MO1-*tbx20*: 86.5±10 SD, n = 10 hearts, 3 independent experiments; p = 0.0001) (Fig 4H). To validate that the reduced number of ventricular cardiomyocytes in Tbx20-ablated embryos was indeed due to impaired proliferation of cardiomyocytes, we additionally performed EdU stainings in MO-control injected embryos and Tbx20 morphants. In fact, we found significantly lower percentages of EdU-positive ventricular cardiomyocytes in MO-*tbx20* injected zebrafish embryos at 72 hpf (MO1-control: 6±2% SD, MO1-*tbx20*: 1±2% SD, n = 10 hearts, 3 independent experiments; p = 0.0001) (Fig 4I–4K), confirming that Tbx20 is an essential driver of cardiomyocyte proliferation in the embryonic zebrafish heart.

### Ectopic expression of wild-type *tbx20* mRNA restores cardiomyocyte proliferation in *whz* mutants

**Fig 5:**
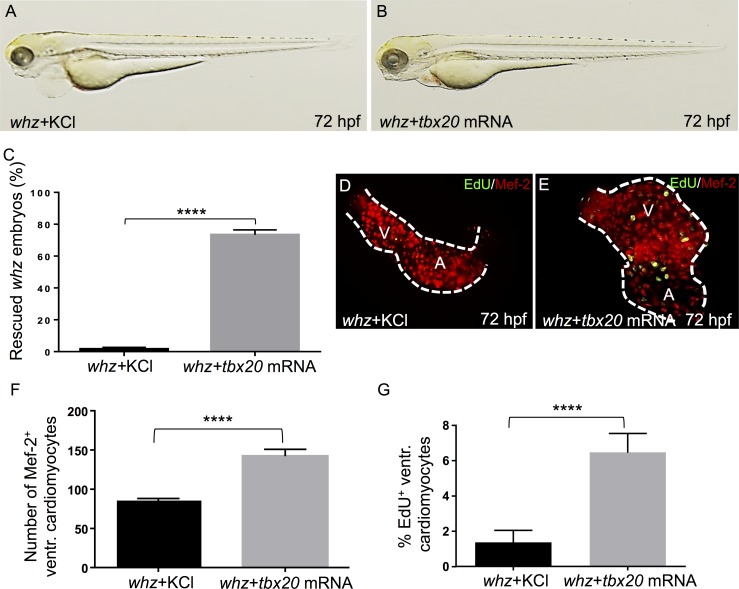
Ectopic expression of wild-type Tbx20 rescues *whz* mutant embryos. **(A, B)** Lateral view of *whz* mutant embryos control-injected with KCl **(A)** and wild-type zebrafish *tbx20* mRNA **(B)**, respectively. **(C)** Ectopic expression of wild-type zebrafish *tbx20* mRNA can rescue the heart phenotype of 73% of homozygous *whz* mutant embryos, whereas injection of KCl has no effect. **(D, E)** Dissected *whz* hearts injected with KCl **(D)** and wild-type *tbx20* mRNA **(E)** are stained against MEF-2 (red) after incorporation of 5-ethynyl-2'-deoxyuridine (EdU; green) to visualize cardiomyocyte proliferation. **(F)** *whz* mutant hearts injected with wild-type *tbx20* mRNA show significantly increased numbers of ventricular cardiomyocytes at 72 hpf **(E)** compared to control-injected *whz* mutants **(D)** (*whz+tbx20* mRNA: 142.5±10 SD, *whz* + KCl: 87.82±10 SD, n = 10; p = 0.0001). **(G)** Ventricular cardiomyocyte proliferation in *whz* mutant embryos injected with *tbx20* mRNA is significantly enhanced compared to control injected mutants at 72 hpf (*whz*+*tbx20* mRNA: 7±3% SD, *whz*+KCl: 1±2% SD, n = 10; p = 0.0001).

To finally prove that impaired cardiomyocyte proliferation in *whz* mutant embryos is indeed caused by loss of Tbx20 function, we next performed a genetic rescue experiment by ectopically expressing wild-type *tbx20* (*tbx20*^*wt*^*)* mRNA in homozygous mutant *whz* embryo. To do so, we injected 8 ng of *tbx20*^*wt*^ into one-cell stage *whz* mutant embryos derived from intercrossing heterozygous *whz* zebrafish (n = 150, 3 independent experiments). As shown in Fig 5, in 73% ± 3.4% of homozygous mutant *whz* embryos, cardiac growth was completely restored by injection of wild-type *tbx20* mRNA (Fig 5A–5C). Cardiomyocyte numbers of *tbx20* mRNA injected *whz* mutant ventricles were comparable to wild-type littermates and significantly increased compared to control-injected *whz* mutants at 72 hpf (*whz + tbx20* mRNA: 142.5±10 SD, *whz* + KCl: 87.82±10 SD, n = 10 hearts, 3 independent experiments; p = 0.0001) (Fig 5F). Furthermore, to validate that the reconstitution of cardiac growth in *tbx20* mRNA injected *whz* mutant was due to re-initiated cardiomyocyte proliferation, we evaluated cell proliferation by EdU stainings in *tbx20* mRNA and control injected *whz* mutant embryos. In fact, we found significantly increased numbers of EdU-positive cardiomyocytes at 72 hpf (*whz* + *tbx20*^*wt*^: 7±3% SD, *whz* + KCl: 1±2% SD, n = 10 hearts, 3 independent experiments; p = 0.0001) (Fig 5D, 5E and 5G), indicating that ectopic expression of wild-type *tbx20* mRNA in *whz* mutant embryos is able to restore cardiomyocyte proliferation.

### Overexpression of Tbx20 in wild-type zebrafish embryos impairs ventricular cardiomyocyte proliferation

**Fig 6:**
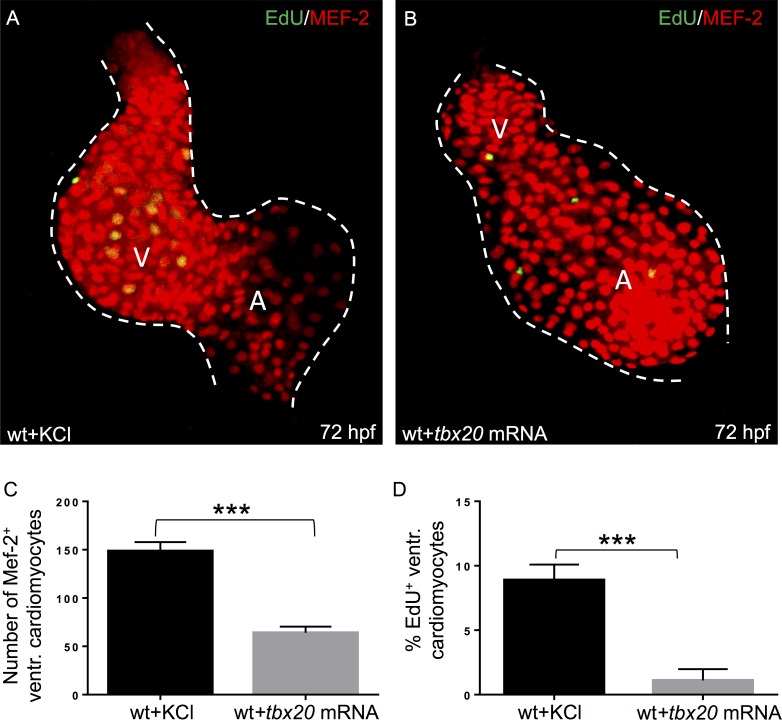
Overexpression of *tbx20* in wild-type zebrafish embryos results in reduced ventricular cardiomyocyte proliferation. **(A, B)** Dissected wild-type hearts injected with KCl **(A)** and *tbx20* mRNA **(B)** at 72 hpf, stained against MEF-2 (red) after incorporation of 5-ethynyl-2'-deoxyuridine (EdU; green) to visualize the effect of *tbx20* overexpression on cardiomyocyte proliferation. **(C)** Wild-type zebrafish hearts injected with wild-type *tbx20* mRNA show significantly reduced numbers of ventricular cardiomyocytes at 72 hpf compared to control injected hearts (wt+KCl: 148.6±2.9; wt + *tbx20* mRNA: 64.0±2.017, n = 10, p< 0.0002). **(D)** Ventricular cardiomyocyte proliferation in wild-type embryos injected with *tbx20* mRNA is significantly reduced compared to control injected embryos at 72 hpf (wt+KCl: 8.9 ± 0.38; wt+*tbx20* mRNA: 1.1±0.28, n = 10, p = 0.0001).

Next, to analyze the impact of ectopically overexpressed *tbx20* mRNA on cardiomyocyte proliferation in a wild-type background, we injected 8 ng wild-type *tbx20* (*tbx20*^*wt*^) mRNA into wild-type zebrafish embryos at the one-cell stage. The effect of Tbx20 overexpression on cardiomyocyte numbers and their proliferation was analyzed by MEF-2 and EdU co-stainings. Interestingly, ventricular cardiomyocyte numbers were significantly reduced in *tbx20* mRNA injected zebrafish embryos compared to KCl control injected littermates at 72 hpf (wt+KCl: 148.6±2.9; wt+*tbx20* mRNA: 64.0±2.0, n = 10 hearts, 3 independent experiments; p<0.0002) (Fig 6A–6C). Quantification of EdU-positive cardiomyocytes 72 hpf showed that cardiomyocyte proliferation was significantly reduced in wild-type embryos overexpressing Tbx20 compared to control injected wild-type littermates (wt+KCl: 8.9±0.38; wt+*tbx20* mRNA: 1.1±0.28, n = 10 hearts, 3 independent experiments; p = 0.0001) (Fig 6D). In summary, our findings imply that Tbx20 activity needs to be tightly regulated during cardiac development to guarantee regular heart growth in zebrafish *in vivo*.

## Discussion

Due to its optical translucency during embryogenesis, the zebrafish is a highly suitable model system to investigate heart development *in vivo*. In particular, the cellular and molecular processes that are involved in embryonic cardiac growth can be easily assessed in zebrafish *in vivo*. In search for novel drivers of embryonic heart growth, we isolated here in a large scale ENU mutagenesis screen the “small heart” zebrafish mutant *weiches herz (whz*) and found that proliferation of ventricular cardiomyocytes is severely reduced in *whz* mutants. By positional cloning, as well as loss- and gain-of function experiments we demonstrated here for the first time that zebrafish Tbx20 is essentially involved in the regulation of embryonic heart growth by orchestrating cardiomyocyte proliferation.

Loss of Tbx20 in *weiches herz* mutant zebrafish embryos results in pathological cardiac hypoplasia. In contrast to zebrafish embryos deficient for the PAF1 complex component Ctr9 or the cardiac sodium channel Scn5Lab [23], we find that cardiac hypoplasia in *whz* is not caused by a reduced amount of cardiac precursor cells in the anterior lateral plate mesoderm. These findings are consistent with data derived from Tbx20 morphant embryos demonstrating that the expression of *nkx2*.*5* in the anterior lateral plate mesoderm at the 12 somite stage is unaltered by the targeted Morpholino-mediated loss of Tbx20 [15]. In fact, by EdU-staining that specifically mark cells in the S-phase of the cell cycle, we found here that embryonic cardiomyocytes in Tbx20-deficient *whz* mutant embryos fail to proliferate, suggesting that Tbx20 might be crucial to control embryonic cardiomyocyte proliferation in zebrafish.

The *whz* mutation in zebrafish Tbx20 is a transversion of the nucleotide Adenine to Cytosine at the stop codon of zebrafish *tbx20*, resulting in a change of the *tbx20* stop codon to the amino acid Cysteine (“nonstop” mutation) and thereby an extension of the Tbx20 protein by 29 amino acids. As shown by Western Blot analyses, this putative protein prolongation results in the destabilization of Tbx20. Tbx20 mRNA levels are unaffected by the nonstop mutation. Similar effects of stop codon mutations on protein stability were observed in other species such as zebrafish, mice and humans [24–27]. As shown in mammalian cells, expression levels of nonstop mRNAs generally appeared unaltered, whereas ribosome stalling at the 3-prime end of the elongated nonstop mRNA interfered with regular translation and led to premature termination of protein synthesis [28–30].

We found here that the *whz* mutation interferes with Tbx20 protein stability resulting in complete loss of Tbx20 function. By Morpholino-mediated knock-down of zebrafish *tbx20* and the subsequent evaluation of cardiomyocyte proliferation we confirmed the loss of function effect of the *whz* mutation. In a previous study, where zebrafish Tbx20 was also partially inactivated by a Morpholino-based antisense oligonucleotide approach, Szeto and co-workers found defective heart chamber morphology and impaired development of the boundaries between both cardiac chambers [15], phenotypic characteristics that were also present in our *whz* mutant embryos, whereas, the impact of loss of zebrafish Tbx20 function on embryonic cardiomyocyte proliferation was not evaluated in this study.

Similar to Tbx20-deficient *whz* mutant embryos, targeted ablation of Tbx20 in mice was recently shown to lead to hypoplastic hearts due to impaired cardiomyocyte proliferation, and thereby embryonic lethality at mid-gestation [13, 14, 31–34]. Cardiomyocyte-specific ablation of Tbx20 in adult mice leads to cardiomyopathies accompanied by arrhythmias, heart failure and ultimately death [31]. Remarkably, cardiomyocyte-specific overexpression of Tbx20 can enhance cardiomyocyte proliferation in fetal ventricular cardiomyocytes leading to increased thickening of the myocardium [35], whereas overexpression of Tbx20 in the embryonic mouse heart at E9.5 resulted in reduced cardiomyocyte proliferation rates, suggesting an opposite impact of Tbx20 overexpression on cardiomyocyte proliferation in embryonic and fetal cardiomyocytes of mice [35]. We found here that, similar to the situation in embryonic mice, overexpression of Tbx20 in the zebrafish embryo resulted in significantly reduced cardiomyocyte numbers due to impaired proliferation, suggesting conserved roles of Tbx20 during embryonic heart growth in zebrafish and mice. Whether Tbx20 overexpression in larval or adult zebrafish leads to reduced or increased cardiomyocyte proliferation rates is currently not known. Interestingly, in adult murine hearts, Tbx20 overexpression beginning in the fetal period promotes the activation of cardiac proliferative pathways such as BMP2/pSMAD1/5/8 and PI3K/AKT/GSK3β/β-catenin signaling, thereby maintaining adult cardiomyocyte in an immature state *in vivo* [35]. Very recently, Xiang et al. were even able to show that Tbx20 overexpression significantly increased cardiomyocyte proliferation and cardiac repair after induced myocardial infarction (MI) [36].

Tbx20 was recently shown to physically bind and thereby repress the cell cycle inhibitory proteins p21 and Meis1 as well as the antiproliferative protein Btg2, thereby activating cardiomyocyte proliferation [36]. Transcriptional profiling of Tbx20-deficient *weiches herz* mutant zebrafish hearts as well as hearts with stable, cardiomyocyte-specific overexpression of Tbx20 will help us to identify the Tbx20-dependent regulatory transcriptional network that controls cardiomyocyte proliferation.
